# A Single-Center Prospective Evaluation of Oncoplastic Crescent Incision in the Surgical Management of Large and Multiple Benign Breast Lesions

**DOI:** 10.7759/cureus.90059

**Published:** 2025-08-14

**Authors:** Suprateek Saha, Swagata Bramhachari, Moorat Singh Yadav, Saif Khan, Radha Sarawagi, Ved Prakash Rao Cheruvu

**Affiliations:** 1 Department of General Surgery, All India Institute of Medical Sciences, Bhopal, Bhopal, IND; 2 Department of Radiodiagnosis, All India Institute of Medical Sciences, Bhopal, Bhopal, IND; 3 Department of Plastic and Reconstructive Surgery, All India Institute of Medical Sciences, Bhopal, Bhopal, IND

**Keywords:** benign breast lesions, cosmetic outcome, crescent oncoplasty incision, oncoplastic breast surgery, patient satisfaction score, single-center study

## Abstract

Background

Benign breast diseases constitute a significant proportion of breast pathologies, often requiring surgical excision. Excision of large or multiple lesions by conventional approaches may compromise cosmetic outcomes, especially for peripheral lesions. Oncoplastic techniques offer a way to achieve complete excision while preserving breast aesthetics. This study aimed to evaluate the efficacy, safety, cosmetic outcomes, and patient satisfaction associated with the oncoplastic crescent incision technique for the excision of large and/or multiple benign breast lesions compared to the conventional periareolar approach.

Methodology

A prospective, comparative cohort study was conducted in a single tertiary care center, from April 2022 to August 2023, on 74 histologically confirmed large (>3 cm) and/or multiple benign breast lesions. Breast lesions were excised by crescent oncoplasty incision (Group A) or periareolar incision (Group B). Surgical outcomes, postoperative complications, scar quality, breast symmetry, patient satisfaction, and patient-reported outcomes were assessed using objective measurements such as the Vancouver Scar Scale, sonographic scar thickness, and the BREAST-Q questionnaire with a follow-up of three months.

Results

The crescent incision provided better access for peripheral lesions (mean lesion-to-nipple-areolar complex (NAC) distance: 3.20 ± 0.71 cm vs. 2.37 ± 0.82 cm, p < 0.001) and was more frequently used for lesions >5 cm (33% vs. 9%, p = 0.0051). Postoperative breast symmetry was significantly better in the crescent group based on sternal notch-to-nipple and midline-to-nipple measurements (p < 0.01). Cosmetic outcomes were superior in the crescent group based on the Vancouver Scar Scale (4.24 ± 1.93 vs. 6.62 ± 1.72, p < 0.001) and sonographic scar thickness (p = 0.02). Nipple sensation was preserved in all Group A patients. BREAST-Q scores for satisfaction, psychosocial, sexual, and physical well-being were comparable between groups throughout follow-up.

Conclusions

The oncoplastic crescent incision is a safe and effective alternative to the conventional periareolar approach for excising large or multiple benign breast lesions. It provides superior cosmetic outcomes, better lesion access, and comparable patient-reported quality of life, supporting its broader adoption in benign breast surgery.

## Introduction

Benign breast disease encompasses a diverse group of non-cancerous conditions, ranging from mild abnormalities to well-defined pathologies, usually presenting as breast lumps, pain, or nipple discharge in women in their second to fourth decades of life. They account for nearly 68% of all breast lesions, with breast lumps being the most common presentation in 80% of cases [1]. Among these, fibroadenoma remains the most frequently encountered benign lesion, particularly in women aged 20-30 years, comprising 77.6% of cases. Less common entities include phyllodes tumors (3.4%), gynecomastia (4.3%), and others [2].

Surgical excision with clear margins remains the standard treatment for palpable benign breast lesions to prevent recurrence. Historically, breast lumps were excised using direct overlying incisions, often resulting in visible scarring and suboptimal cosmetic results [3]. With an increasing focus on surgical cosmesis, now aesthetically placed, hidden scars in the axilla, inframammary fold, and periareolar region are the preferred options [4].

The conventional periareolar incision offers good cosmetic results for small breast lesions due to its hidden scar, but becomes inadequate for large or multiple lesions such as giant fibroadenomas or phyllodes tumors. Wide periareolar incisions (more than half of the areola) in such cases pose risks of limited access and compromised nipple-areolar vascularity [5], and achieving clear margins often results in parenchymal loss and breast deformity and asymmetry, requiring immediate reconstruction [6]. Poor cosmetic outcomes from benign lesion excision can significantly impact psychological well-being and quality of life, especially in younger patients who are sensitive to cosmetic outcomes [4]. To overcome these limitations of conventional incisions, oncoplastic breast surgery, which was traditionally used in breast-conserving cancer surgery, is now being used for excising large or multiple benign breast lesions [7].

Oncoplastic breast surgery combines oncologic safety with plastic surgery reconstructive techniques to allow complete excision of large lesions with clear margins and better cosmesis by immediate reconstruction of the parenchymal defect using either volume displacement or volume replacement oncoplastic techniques. Volume displacement reshapes the breast by tissue redistribution and is classified as level 1 (<20% volume excised) or level 2 (20-50%) [8]. Common level 1 techniques, such as round block, crescent, batwing, and hemi-batwing incisions, provide adequate access while minimizing scarring and preserving breast shape and function [9].

The crescent oncoplastic technique is a level 1 approach ideal for excising large or multicentric benign lesions, especially in the upper outer quadrants above the areola, while preserving the nipple and correcting mild ptosis. It offers hidden scarring, clear margins, preserved nipple sensation, and maintained contour, ensuring excellent cosmetic outcomes and patient satisfaction [9]. Despite these advantages, its use remains underreported in current literature.

This study assesses the effectiveness of the oncoplastic crescent incision compared to conventional periareolar incisions for the excision of large (≥3 cm) and multiple benign breast lesions, focusing on surgical and cosmetic outcomes, patient satisfaction, and quality of life to determine the most suitable surgical approach for such cases.

## Materials and methods

Study design and setting

This prospective, comparative cohort study was conducted at a tertiary care institute in Central India from April 2022 to August 2023, following approval from the Institutional Ethics Committee, All India Institute of Medical Sciences, Bhopal (approval number: LOP-IHEC-PGR/2021/PG/Jul/11).

Inclusion and exclusion criteria

Patients aged 18-60 years of either sex with benign breast lesions measuring ≥3 cm or multiple lesions involving >1 quadrant of the same breast who were scheduled for excision were included in this study. Patients with suspected malignancy, prior breast surgery/radiotherapy, nonpalpable lesions, psychiatric comorbidities, or those who declined consent were excluded from the study.

Sample size

Convenience sampling was used for sample size estimation. In total, 74 participants were enrolled as per the inclusion criteria. All eligible participants (n = 74) were enrolled in the study after obtaining written informed consent. Preoperative demographic and clinico-radiological data were collected for all participants and documented in the case record form.

Group allocation and interventions

At the investigator’s discretion, participants were evenly divided into two groups (n = 37 participants each), undergoing excision as follows: Group A = oncoplastic crescent incision, and Group B = conventional periareolar incision.

Intervention

Group A (Oncoplastic Crescent Incision)

**Figure 1:**
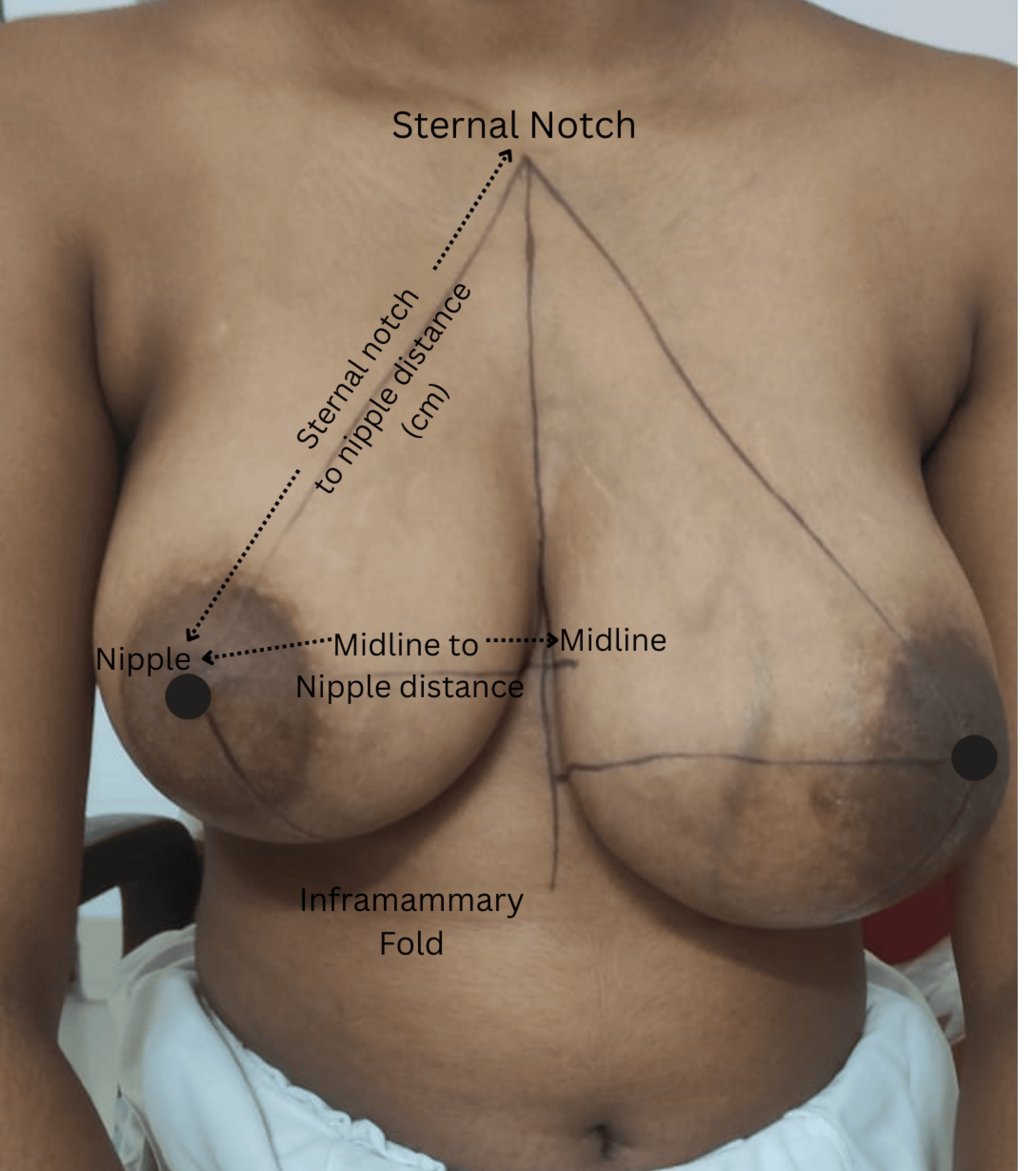
Breast symmetry: preoperative marking of measurements.

Preoperatively, in the erect position, the measurements of the midline-to-nipple, sternal notch-to-nipple, and inframammary fold (IMF)-to-nipple distances were marked and recorded for breast symmetry (Figure 1).

**Figure 2:**
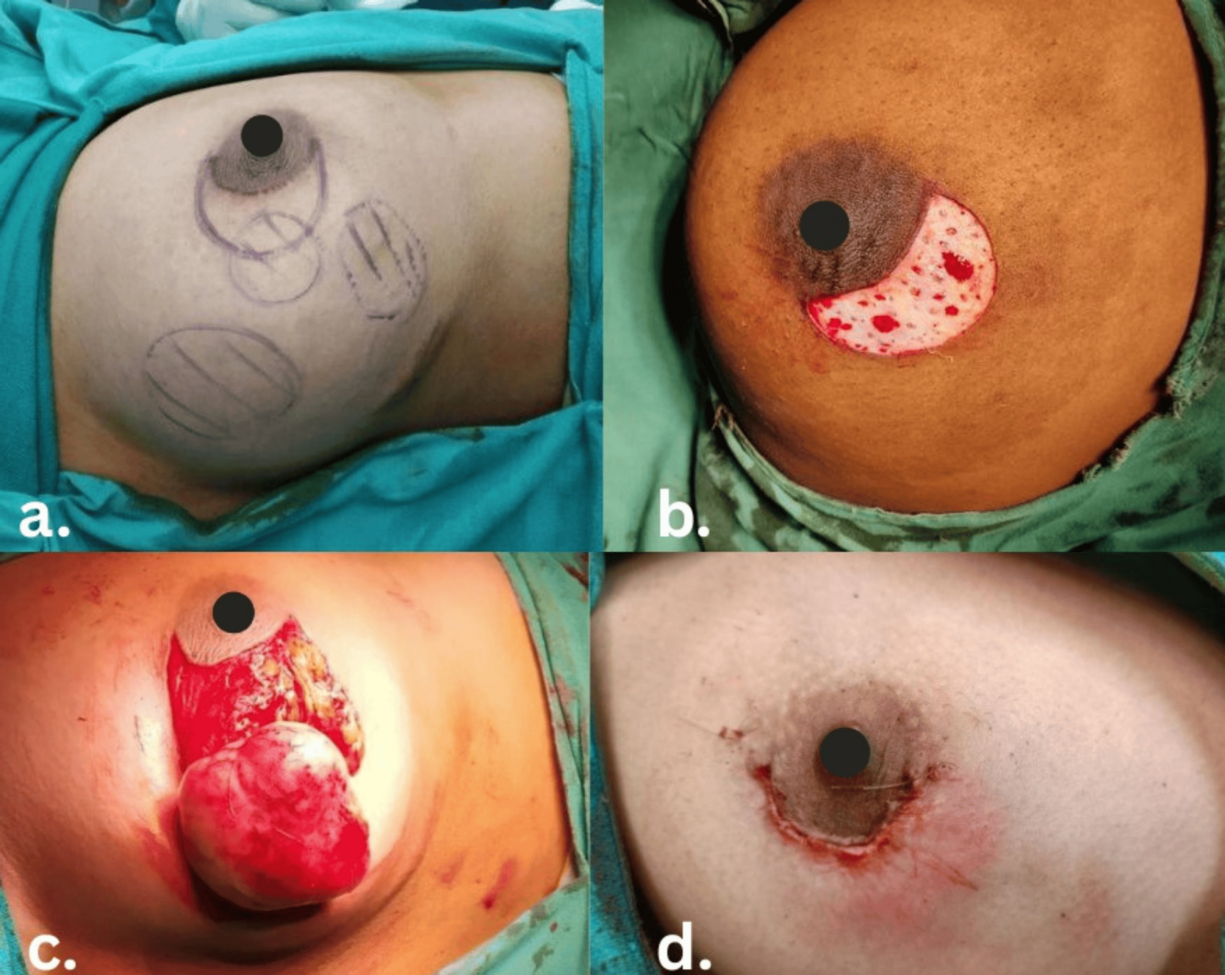
Crescent oncoplasty incision technique. (a) Preoperative marking of the tumor and incision. (b) De-epithelialization between the inner and outer crescent lines. (c) Lumpectomy in process. (d) Closure of the crescent incision by subcuticular suturing.

After marking the boundaries of the lesion and nipple-areolar complex (NAC), a crescent-shaped incision with defined outer and inner lines was marked accordingly (Figure 2a). The dimensions of the crescent incision are tumor-dependent and not based on fixed ratios to the areola. The areola serves as a guide for incision placement, with the inner periareolar “C”-shaped incision limited to a maximum length less than or equal to the areolar diameter and a width of 1.5-2 cm between parallel lines. Under anesthesia, the skin area between the two semi-parallel “C”-shaped lines of the marked crescent incision was de-epithelialized (Figure 2b). The lump was excised with an adequate margin by deepening the superior border of the crescent incision (Figure 2c). With dual-plane mobilization of the breast tissue, parenchymal approximation at the resection site was done by absorbable 3-0 polyglactin 910 sutures, thereby minimizing postoperative contour deformities. The skin flaps were then meticulously approximated following the insertion of de-epithelialized tissue beneath and sutured with 3-0 polyglecaprone subcuticular sutures (Figure 2d) [9].

**Figure 3:**
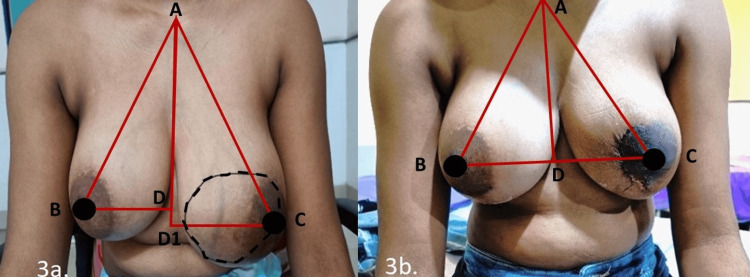
Symmetry and cosmesis. (a) Preoperative lump marking. (b) Postoperaive oncoplastic crescent incision scar. Measurements: AB = sternal notch to right nipple; AC = sternal notch to left nipple; DB = midline to right nipple; D1C = midline to left nipple (preoperative); DC = midline to left nipple (postoperative)

Postoperative cosmetic outcomes were assessed through breast symmetry and scar evaluation. Breast symmetry was measured objectively by determining the distances from the sternal notch and midline to each nipple, forming a triangular reference (Figure 3). Scar cosmesis was evaluated subjectively by the patients using a scar self-rating scale. Objective scar assessment included sonographic measurement of scar thickness and evaluation with the Vancouver Scar Scale, performed independently by a co-investigator (plastic surgeon) who was not in the operative team. Patient-reported outcome was assessed using the Breast Q questionnaire.

Group B (Conventional Periareolar Incision)

**Figure 4:**
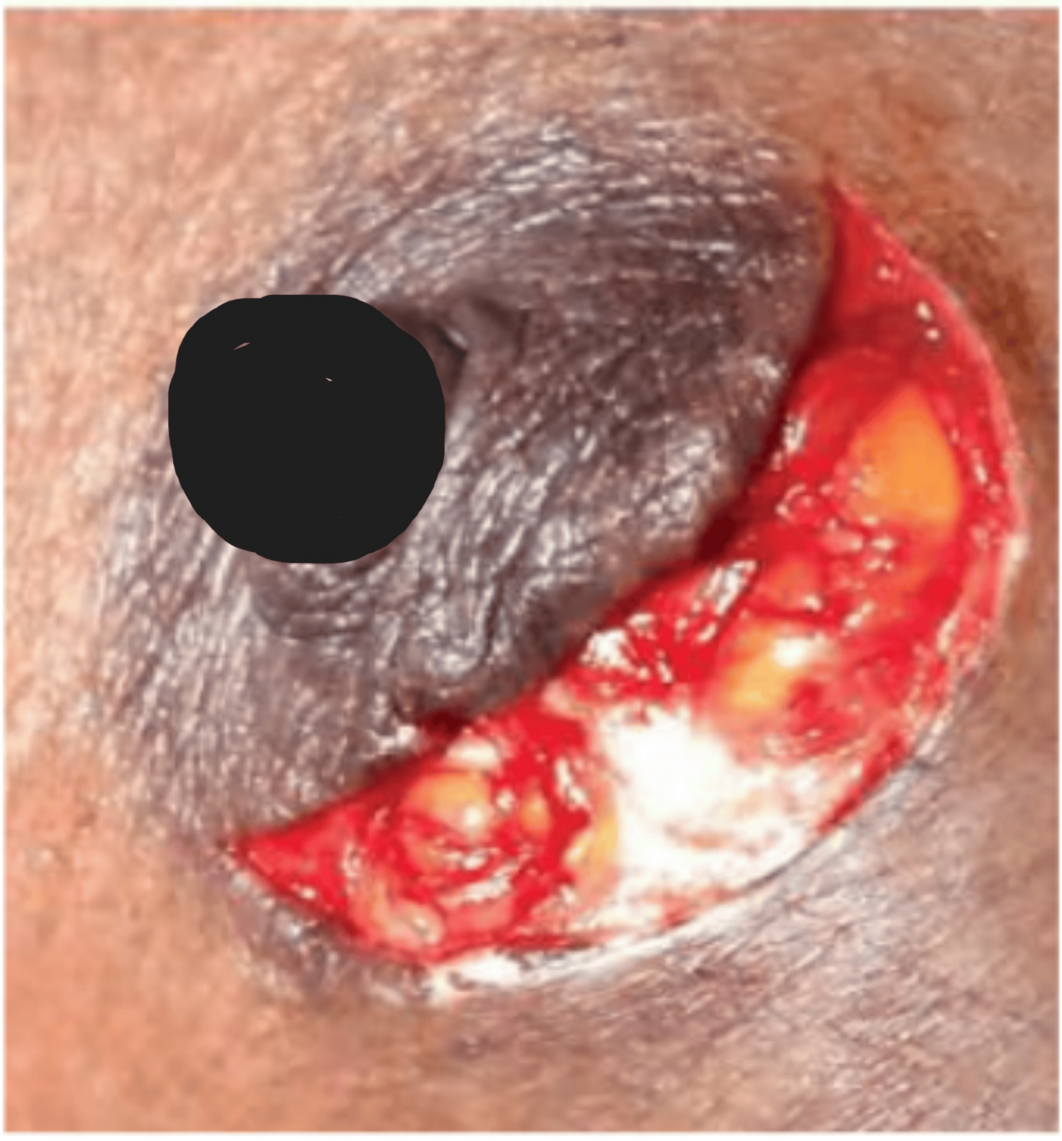
Periareolar incision.

A curvilinear periareolar incision (≤50% areolar circumference) was marked, and the lesion was excised from the breast parenchyma with or without mild subcutaneous tunneling, depending on the position of the lump. Closure was done by 3-0 polyglactin 910 subcutaneous sutures and 3-0 polyglecaprone subcuticular sutures [6] (Figure 4).

Outcome Measures

The study outcome measures are detailed in Table 1.

**Table 1:** Outcome measures.

Domain	Measure/Tool	Details	Timing
Breast symmetry	Manual measurements	Midline-to-nipple, sternal notch-to-nipple, inframammary fold (IMF)-to-nipple (Figure 1)	Preoperative and postoperative
Pain	Visual Analogue Scale (VAS)	Scale of 1–10 [10]	Postoperative
Complications	Clavien-Dindo Classification	Grading of postoperative complications [11]	Postoperative
Surgical site morbidity	Clinical assessment	Discharge (serous, purulent, hemorrhagic); swelling (hematoma, seroma, edema)	Postoperative
Nipple sensation	Graded 0–3 by stimulus	Touch (cotton wick), pressure (needle cap), pain (needle prick)	Postoperative
Cosmetic outcomes	a) Scar Self-Rating Scale;	a) Subjective rating [12]	Postoperative
	b) Vancouver Scar Scale;	b) Vascularity, pigmentation, pliability, height, pain, itchiness [13]	
	c) Sonography	c) Scar thickness via a 15 MHz probe	
Quality of life by patient-reported outcome measures (PROM)	BREAST-Q Questionnaire	Psychosocial, sexual, physical well-being; satisfaction domains (score: 1–100) [14]	Preoperative and postoperative

Follow-up protocol

**Figure 5:**
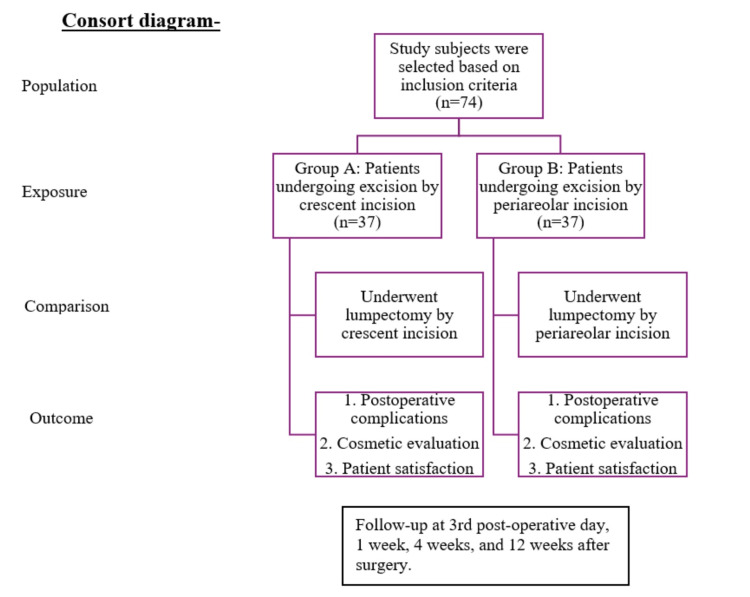
CONSORT flowchart.

All participants were evaluated at postoperative days three and seven and weeks four and twelve for clinical outcomes, complications, and quality of life metrics. The CONSORT flowchart of the study is shown in Figure 5.

Statistical analysis

Data were collected using a standardized proforma and entered into Microsoft Excel (Microsoft Corp., Redmond, WA, USA), followed by data cleaning. Categorical variables were expressed as frequencies and proportions, and continuous variables as mean ± standard deviation. Intergroup comparisons were conducted using the chi-square test for categorical variables and the unpaired t-test for continuous variables. A p-value <0.05 was considered statistically significant. Analysis was done using SPSS (IBM Corp., Armonk, NY, USA).

## Results

In total, 74 patients with benign breast lesions measuring ≥3 cm were enrolled and equally assigned to two groups: 37 underwent excision by the oncoplastic crescent incision (Group A), and 37 by the conventional periareolar incision (Group B). The mean age of the patients in group A was 27.14 ± 8.64 years, and in group B was 34.49 ± 11.99 years (p = 0.01). Fibroadenoma was the most common benign lesion (n = 68, 91.89%), followed by gynecomastia (n = 3, 4.0%) and benign phyllodes tumor (n = 2, 2.7%). Of the 74 participants, three were males who underwent excision of lesions via the crescent incision. The pattern of distribution of the breast lesions was comparable in both groups, with the upper outer quadrant being the most frequently involved (n = 21, 56.8%) (Table 2).

**Table 2:** Demographic data of the study participants. Values are presented as the number of patients (percentage). Statistical analysis was conducted using the chi-square test (χ²) for categorical variables. The test statistic column displays the χ² value for each comparison. P-values <0.05 were considered statistically significant.

Variable	Crescent group (n = 37)	Periareolar group (n = 37)	Test statistic	P-value
Sex			χ² = 3.16	0.242
Female	34 (91.9%)	37 (100.0%)		
Male	3 (8.1%)	0 (0.0%)		
Site of involvement			χ² = 13.41	0.099
Upper outer quadrant	21 (56.8%)	21 (56.8%)		
Upper inner quadrant	5 (13.5%)	4 (10.8%)		
Lower outer quadrant	0 (0.0%)	5 (13.5%)		
Lower inner quadrant	5 (13.5%)	7 (18.9%)		
Central quadrant	1 (2.7%)	0 (0.0%)		
Lower inner + lower outer	1 (2.7%)	0 (0.0%)		
Upper outer + lower outer	1 (2.7%)	0 (0.0%)		
Involving all quadrants	3 (8.1%)	0 (0.0%)		

The size of breast lesions ranged from 3 to 9 cm, with an average lump size in group A of 3.97 × 3.22 cm, whereas in group B, it was 3.22 × 3.08 cm (p = 0.026). The excision of benign breast lesions larger than 5 cm was performed by the crescent oncoplastic incision, which provides better access and management for larger tumors. The mean distance of the lump from NAC in Group A (3.20 ± 0.71 cm) and Group B (2.37 ± 0.82 cm) was statistically significant (p < 0.001), indicating that the crescent oncoplastic incision provides better access for the excision of peripheral lesions than periareolar incision.

The mean preoperative midline-to-nipple distance (8.47 ± 4.21 cm) and postoperative midline-to-nipple distance (8.80 ± 2.53 cm) crescent group, in comparison to the mean preoperative midline-to-nipple distance (6.15 ± 3.45 cm) and postoperative midline-to-nipple distance (8.44 ± 2.45 cm), were significant (p < 0.01), implying that the nipple-areola repositioning and symmetry were better in the crescent group. Similarly, the mean preoperative (22.57 ± 3.12 cm) and postoperative (21.10 ± 3.05 cm) sternal notch to nipple distance in the crescent group was significantly better (p < 0.01) than the mean preoperative (22.59 ± 1.72 cm) and postoperative distance (21.80 ± 1.65 cm) in the periareolar group, indicating that the crescent incision performed significantly better compared to the periareolar incision (p < 0.01) in terms of achieving symmetry between the two breasts after excision of the lump (Table 3).

**Table 3:** Preoperative and postoperative measurements. Values are presented as mean ± SD or n. P-values <0.05 were considered statistically significant. Statistical tests used: chi-square test (χ²) for categorical comparisons. Independent t-test (t) for continuous intergroup comparisons. Paired t-test (†) for intragroup (pre-post) comparisons.

Variables		Crescent group (N = 37)	Periareolar group (N = 37)	*P*-value
Lump size	<5 cm	25 (67%)	31 (91%)	
	≥5 cm	12 (33%)	6 (9%)	
	Multiple lumps in more than 1 quadrant	4 (10.8%)	0 (0%)	
	Average lump size (cm)	3.97 × 3.22	3.22 × 3.08	0.026
Distance from nipple-areolar complex (cm), mean ± SD		3.20 ± 0.71	2.37 ± 0.82	<0.001
Midline-to-nipple distance (cm)	Preoperative (mean ± SD)	8.47 ± 4.21	6.15 ± 3.45	
	Postoperative (mean ± SD)	8.80 ± 2.53	8.44 ± 2.45	
	Intragroup p-value	<0.01	0.08	
Sternal notch-to-nipple distance (cm)	Preoperative (mean ± SD)	22.57 ± 3.12	22.59 ± 1.72	
	Postoperative (mean ± SD)	21.10 ± 3.05	21.80 ± 1.65	
	Intragroup p-value	<0.01	0.06	

The postoperative pain was assessed using the Visual Analog Scale VAS score, which was comparable in both groups, but the mean value in Group A (1.08 ± 1.18) was more than in Group B (0.51 ± 0.73) after one week, though statistically not significant. During the postoperative period, one patient in Group A and three patients in Group B had serous discharge from the wound. Nipple sensations were preserved in all patients in both groups. The Clavein-Dindo grading in both groups was comparable, ranging from group 2 to group 3A (Table 4).

**Table 4:** Postoperative complications. Values are presented as mean ± SD for continuous variables and n for categorical variables. *: P-values <0.05 were considered statistically significant. Statistical tests used: Independent t-test (t) for Visual Analog Scale (VAS) pain scores. Chi-square test (χ²) for categorical outcomes (discharge, complications). When counts were very low (e.g., all zeros), Fisher’s exact test was applied or marked with “—” when the test statistic was not applicable.

Variable	Crescent group (n = 37)	Periareolar group (n = 37)	Test statistic	P-value
VAS Score (mean ± SD)				
Day 3	3.46 ± 1.44	3.43 ± 1.50	t = 0.07	0.94
1 week	1.08 ± 1.18	0.51 ± 0.73	t = 2.21	0.03*
4 weeks	0.03 ± 0.16	0.03 ± 0.16	t = 0.00	1.00
12 weeks	0 ± 0	0 ± 0	—	1.00
Discharge, n (%)	1 (2.7%)	3 (8.1%)	χ² = 0.52	0.61
Swelling, n (%)	0 (0%)	0 (0%)	—	1.00
Presence of Nipple Sensation	37 (100%)	36 (97.3%)	χ² = 1.00	1.00
Clavien-Dindo Complication Grade				
Day 3				
Grade II	1 (2.7%)	3 (8.1%)	χ² = 1.71	0.19
Grade III A	0 (0%)	2 (5.4%)		
1 week				
Grade II	2 (5.4%)	2 (5.4%)	χ² = 0.04	0.84
Grade III A	1 (2.7%)	2 (5.4%)		
4 weeks				
Grade II	0 (0%)	2 (5.4%)	χ² = 0.47	0.49

Cosmetic assessment of the scar was done subjectively by the patient’s Scar Self-Rating Scale, which was comparable (p < 0.01) between groups, and objectively by the Vancouver Scar Scale. The score in Group A (4.24 ± 1.93) was found to be statistically significant (p < 0.001) compared to Group B (6.62 ± 1.72), showing superior cosmesis by crescent oncoplastic incision. High-resolution ultrasonography for scar thickness was comparable (p = 0.02) (Table 5).

**Table 5:** Comparison of scar-related scores. Values are reported as mean ± standard deviation. Intergroup comparisons were performed with the independent samples t-test (df = 72). P-values <0.05 were considered statistically significant.

Scale (mean ± SD)	Crescent group	Periareolar group	Test statistic	P-value
Scar Self-Rating Scale	1.35 ± 0.48	2.05 ± 1.26	*t* = ‑3.16	<0.01
Vancouver Scar Scale	4.24 ± 1.93	6.62 ± 1.72	*t* = ‑5.60	<0.001
High-resolution ultrasonography for scar thickness	2.24 ± 0.64	2.78 ± 1.18	*t* = ‑2.45	0.02

Patient satisfaction and quality of life were assessed preoperatively and postoperatively by patient-reported outcomes (BREAST-Q). Patient satisfaction regarding cosmesis, breast symmetry, and surgical outcomes was comparable preoperatively and postoperatively (1, 4, and 12 weeks) in both groups. The quality of life as reported by patients regarding psychosocial well-being, sexual well-being, and physical well-being of the chest and upper body was also found to be comparable in both groups pre- and postoperatively and in the follow-up period up to 12 weeks (Table 6).

**Table 6:** Comparison of BREAST-Q scores between the two groups Scores represent BREAST-Q 2.0 domain-specific patient-reported outcomes. Comparisons between groups were performed using the independent samples t-test. P-values <0.05 were considered statistically significant. No statistically significant differences were observed across domains or time points between groups.

Domain	Time point	Crescent group (mean ± SD)	Periareolar group (mean ± SD)	Test statistic	P-value
Satisfaction with breasts	Preoperative	52.1 ± 1.0	51.9 ± 1.2	t = 0.23	0.81
	1 week	95.2 ± 2.2	94.1 ± 2.9	t = 0.78	0.44
	4 weeks	96.3 ± 1.8	95.6 ± 2.5	t = 0.49	0.63
	12 weeks	97.0 ± 1.6	96.3 ± 2.7	t = 0.74	0.46
Psychosocial well-being	Preoperative	48.7 ± 0.9	49.0 ± 1.1	t = –0.29	0.77
	1 week	91.3 ± 2.5	90.8 ± 3.1	t = 0.65	0.52
	4 weeks	92.0 ± 2.1	91.7 ± 2.8	t = 0.52	0.61
	12 weeks	92.6 ± 2.2	91.9 ± 2.6	t = 0.68	0.50
Physical well-being	Preoperative	49.1 ± 0.8	48.9 ± 1.0	t = 0.15	0.88
	1 week	91.7 ± 2.6	91.0 ± 3.0	t = 0.71	0.48
	4 weeks	92.5 ± 2.0	92.0 ± 2.7	t = 0.44	0.66
	12 weeks	93.2 ± 2.1	92.5 ± 2.8	t = 0.73	0.47
Sexual well-being	Preoperative	50.0 ± 1.1	49.9 ± 1.2	t = 0.21	0.83
	1 week	93.8 ± 2.4	93.4 ± 2.9	t = 0.69	0.49
	4 weeks	94.5 ± 1.9	94.2 ± 2.6	t = 0.47	0.64
	12 weeks	93.8 ± 2.1	94.5 ± 2.4	t = –0.63	0.53

Excision of large and multiple benign lesions by the crescent oncoplastic incision results in excellent cosmetic outcome and symmetry (Figure 3). It also results in good patient satisfaction regarding cosmesis and overall surgical outcome, along with a good quality of life postoperatively in comparison to the periareolar group.

## Discussion

Benign breast diseases constitute a significant proportion of breast pathologies encountered in clinical practice. Among these, fibroadenoma is the most common, reported in 77.6% of cases, while less frequent lesions include phyllodes tumors (3.4%), gynecomastia (4.3%), and others [2]. A similar finding was found in our study, where fibroadenoma was the predominant lesion (91.89%), with gynecomastia and phyllodes tumor reported in 4% and 2% of cases, respectively.

Benign breast lesions generally range from 2 to 10 cm, with prior studies reporting 53% measuring 2-5 cm, 32% 6-10 cm, and 6% as multicentric, located mostly in the upper half of the breast [15]. In our cohort of lesions ≥3 cm, 69% of lesions are in the upper half of the breast, 75.67% measured 3-5 cm, 24.32% were >5 cm, and 10.8% were multicentric. In this observational study, larger lumps (>5 cm) were excised by crescent incision (33%) compared to periareolar incision (9%). Surgical excision remains a standard treatment option, particularly for large or multiple lesions.

Oncoplasty breast surgery is the integration of breast oncosurgery and plastic surgery, with level 1 oncoplasty being a volume displacement technique, where the available parenchyma and skin are rearranged to restore a symmetrical breast mound. Crescent oncoplasty is a level 1 oncoplasty technique that facilitates margin clearance while optimizing cosmetic outcomes, preserving breast symmetry, and minimizing morbidity, but it has been less studied. In our study, 12 (33%) giant and four (10.8%) multiple benign lesions were excised by the crescent incision with excellent cosmetic outcomes, despite a lack of prior data on this technique.

Periareolar incisions are associated with risks of nipple necrosis, sensory changes, and collapse of the NAC [3,16], especially in young patients, due to the extensive tunneling required for excision of large or peripheral lesions [5]. However, in this study, there was only wound discharge in 8.1% of the periareolar cases compared to 2.7% of the crescent cases. There was no nipple necrosis and no loss of nipple sensations in both groups.

Postoperative pain was found to be slightly higher in the crescent group at the one-week follow-up, but it was transient and resolved with standard analgesics. Crescent incisions enabled better access to peripheral lesions, reflected by a greater mean NAC-to-lesion distance (3.20 ± 0.71 cm vs. 2.37 ± 0.82 cm). It is possible to avoid nipple necrosis and damage to the nipple blood supply by making incisions that do not touch the areola’s border [16].

In phyllodes tumor excision, oncoplastic techniques enable adequate margin clearance (>1 cm) while preserving cosmesis, thereby potentially reducing recurrence and avoiding mastectomy. Some studies have reported the use of the round block oncoplasty technique for the wide local excision of benign phyllodes tumors [15,17,18], but the crescent oncoplasty incision has not been previously reported for the excision of benign phyllodes tumors. In this study, two cases were successfully excised by the crescent oncoplasty incision with oncologically safe margins, without recurrence, and with excellent cosmetic outcomes during 18 months of follow-up.

Breast cosmesis and symmetry after breast oncoplasty surgery are assessed subjectively (patient perception) or objectively by breast measurements on both sides, such as sternal notch-to-nipple (19-24 cm) and nipple-to-midline (8-12 cm) distances forming the “aesthetic triangle” [19], with nipple position at the lateral angles of the base of the triangle representing optimal breast aesthetics [20]. The crescent incision group demonstrated significantly better (p < 0.01) postoperative nipple positioning and breast symmetry compared to the periareolar group. As immediate breast symmetry was achieved, contralateral symmetrization was not required in any cases of large tumors. In benign breast lesions, patients do not consent to contralateral symmetrization; hence, oncoplastic procedures are the best choice.

Cosmetic assessment of the scar using the Self-Scar Rating Scale showed comparable results (p < 0.01), while the Vancouver Scar Scale (p < 0.001) and sonographic scar thickness (p = 0.02) demonstrated a significant advantage for the crescent incision. These findings underscore its aesthetic and safety benefits in large and multiple benign lesion excision [12,13]. No studies have evaluated the crescent oncoplastic scar objectively for scar cosmesis.

The BREAST-Q (Version 2.0), a validated patient-reported outcome measure for breast surgery, was administered pre- and postoperatively. It revealed comparable results between the two incision groups across satisfaction, psychosocial, physical, and sexual well-being domains [14]. Therefore, crescent incision did not negatively impact quality of life compared to periareolar incision.

In the present study, 12 (33%) giant and four (10.8%) multiple benign lesions were excised by the crescent incision with excellent cosmetic outcomes, despite a lack of prior data on this technique. Therefore, the crescent incision appears to be a safe and esthetically favorable standalone technique for the excision of benign lesions situated in the upper half of the breast in mildly ptotic breasts [21].

This study has several limitations. It is a single-center study with a small sample size, limiting the generalizability. There is a potential selection and assessment bias due to the lack of randomization and blinding. The 12-week follow-up may not reflect long-term cosmetic outcomes or patient satisfaction. However, the study offers valuable preliminary data, and the consistent methodology supports its internal validity. These findings provide a foundation for future randomized studies with extended follow-up and cost-benefit analysis.

This study supports the crescent incision as a safe, effective, and cosmetically superior technique for excising large or multiple benign breast lesions. Further multicentric studies with larger cohorts are needed to validate these findings and develop standardized surgical protocols for the excision of large and multiple benign breast lesions.

## Conclusions

The study supports the crescent oncoplastic incision as a safe, effective, and cosmetically superior technique for excising large (≥3 cm) or multiple benign breast lesions. It offers better immediate reshaping for breast symmetry and fewer complications than the conventional periareolar approach, with comparable patient satisfaction and quality of life. The findings advocate for extending oncoplastic principles to benign breast surgery, particularly in mildly ptotic breasts, and highlight the need for larger multicenter studies to standardize surgical protocols. It is particularly effective in challenging cases such as giant fibroadenomas, multicentric lesions, and recurrent phyllodes tumors, offering both adequate tumor excision and immediate restoration of breast contour. Given its versatility and effectiveness, the crescent technique should be regarded as a key surgical option in the treatment of large or recurrent benign breast lesions.
